# Dynamic muscle damage monitoring in pig crush injury: T2-weighted Dixon and 2D ultrasound applications

**DOI:** 10.3389/fvets.2026.1692050

**Published:** 2026-02-25

**Authors:** Guangda Wang, Jiayi Wang, Dou Li, Qi Wang, Zikuo Zhao, Rongbang Chen, Qi Lv, Haojun Fan

**Affiliations:** 1 Institute of Disaster and Emergency Medicine, Tianjin University, Tianjin, China; 2 Department of Computed Tomography and Magnetic Resonance Imaging, The Fourth Hospital of Hebei Medical University, Shijiazhuang, Hebei, China

**Keywords:** crush injury, muscle damage assessment, porcine model, T2-weighted Dixon, ultrasonography

## Abstract

**Background:**

Crush injury (CI) involves compressive trauma causing muscle swelling, compartment syndrome, and neurological damage. We examined T2-Dixon and ultrasound for CI evaluation in pigs, integrating imaging with lab and tissue findings.

**Methods:**

Twelve 15–16-month-old Bama miniature pigs were randomly divided into three extrusion groups: Group A (4 h), Group B (8 h), and Group C (16 h), using custom equipment. Blood samples were collected at baseline (T0), decompression (T1), and 12 h (T2), 24 h (T3), and 72 h (T4) post-decompression. MRI and ultrasound were performed at each time point. At T4, pigs were euthanized, and compressed muscles underwent HE staining for pathological assessment.

**Results:**

Following decompression, Creatine Kinase (CK), Lactate dehydrogenase (LDH), and K^+^ levels initially rose, then declined across all groups. CK peaked at T2 or T3 (*p* < 0.05), with Group B > Group A at T3/T4, and Group C > Group A at T1–T3 (*p* < 0.05). LDH peaked at T2/T3, with Groups B and C > Group A (*p* < 0.05). K^+^ peaked at T1/T2, with Group C showing a significant increase (*p* < 0.05) but no difference between Groups A and B. T2-weighted signal values rose then fell in Groups A/B but increased continuously in Group C, peaking at T2/T3 (*p* < 0.05). Group B > Group A at T2/T3; Group C > Group A at T1–T3 and > Group B at T3 (*p* < 0.05). CK and LDH correlated positively with T2 signal values (strongest in Group C), while K^+^ showed no correlation. Ultrasound revealed mildly enhanced echogenicity and structural disorganization in Group A. Groups B and C showed more severe damage, featuring a homogeneous, featureless appearance with complete loss of normal muscle architecture and heterogeneous echogenicity with sieve-like hypoechogenicity and fat layer edema, respectively. HE staining at T4 demonstrated progressively worse muscle damage with longer extrusion times.

**Conclusion:**

This study confirmed the viability of the CI model through biochemical verification and demonstrated that T2-Dixon and ultrasound synergistically assessed muscle damage, providing a more thorough evaluation that surpasses the constraints of biochemical markers alone.

## 1 Introduction

Crush injury (CI) represents a life-threatening trauma frequently encountered during natural disasters (earthquakes, stampedes) and high-impact accidents, where prolonged mechanical compression, often exceeding 4 h, induces severe muscle necrosis and neurological impairment through ischemic and reperfusion pathways (1–3). Unlike superficial wounds, CI typically presents with a characteristic triad of subcutaneous bruising, cyanosis, and progressive swelling despite deceptively intact skin, a phenomenon termed “the iceberg effect” in trauma medicine. The injury mechanism involves both direct compressive forces and subsequent metabolic cascades, including intracellular calcium overload and mitochondrial dysfunction. Clinical data from mass casualty events consistently show lower extremities are most vulnerable (74% of cases), particularly the calf muscles and thighs, followed by upper limbs (10%, especially forearm compartments) and torso (9%, predominantly crush asphyxia cases) (4, 5). This anatomical distribution reflects both the exposed positioning of limbs during structural collapses and the differential tissue tolerance to ischemic conditions. The delayed presentation of systemic complications, sometimes 24–72 h post-decompression, further complicates early clinical assessment and intervention strategies.

During prolonged muscle compression, progressive myocyte membrane damage increases cellular permeability, allowing sodium/calcium influx that induces edema. Persistent compression may trigger acute compartment syndrome, compromising limb perfusion and neurological function (6, 7). Calcium overload further activates proteolytic enzymes, causing sustained muscle contraction, ATP depletion, and ischemic injury, key steps in rhabdomyolysis (RM) (8). Even post-decompression, ischemia–reperfusion injury exacerbates tissue damage (9). Critically, CI’s systemic effects include hypovolemic shock, acute kidney injury (AKI), electrolyte imbalances (hyperkalemia/hypocalcemia), metabolic acidosis, and multi-organ dysfunction (6, 10–12), drastically increasing treatment complexity and mortality risk. Early diagnosis is thus vital for targeted intervention to halt progression and improve outcomes.

Traditional clinical diagnosis of crush injury (CI) relies on comprehensive history-taking, clinical observation, and laboratory tests. However, the systemic effects of crush syndrome (CS) often lead to nonspecific clinical manifestations (13). Among laboratory indicators, elevated creatine kinase (CK) remains one of the most diagnostic biomarkers (14). While CK and other biomarkers aid disease recognition (15, 16), they cannot precisely localize injuries or objectively assess severity. Recent advances in medical imaging have established its critical role in CI diagnosis, as demonstrated in mass casualty events like the Itaewon stampede (17), the Turkey-Syria earthquake (7), and the Wenchuan earthquake (18). Ultrasound has emerged as a valuable screening tool due to its portability and real-time imaging capabilities, enabling clear visualization of muscle damage, particularly useful for asymptomatic patients (19). Meanwhile, magnetic resonance imaging (MRI) provides superior soft tissue characterization through high-resolution, multiparametric imaging, making it indispensable for both diagnosis and prognostic evaluation of soft tissue injuries (20–22).

This study develops standardized porcine hindlimb crush models to systematically evaluate T2-weighted Dixon sequences and ultrasound. The integrated biochemical and pathological analysis specifically assesses their sensitivity and specificity in detecting injury severity gradients and temporal progression patterns. Beyond enabling accurate severity assessment and prognostic prediction, this multimodal approach provides an evidence-based foundation for clinical strategy development. It also establishes standardized imaging benchmarks that address current diagnostic limitations, particularly the need for precise localization and dynamic monitoring of muscle damage. By comparing these complementary techniques (Dixon’s superior soft-tissue contrast versus ultrasound’s rapid deployability), the study advances evidence-based protocols that can optimize time-sensitive treatment decisions and improve overall patient outcomes in crush injury management.

## 2 Materials and methods

### 2.1 Animal studies and experimental preparation

Twelve conventional (CV) male Bama miniature pigs (15–16 months old, 25 ± 5 kg) were obtained from Beijing Long’an Experimental Animal Breeding Center [License: SCXK (Beijing) 2021-0014]. The animals were housed individually in temperature-controlled, ventilated cages with ad libitum access to food and water. Following a 3-day acclimatization period, food and water were withheld for 24 h before experiments. This study was approved by the Animal Care and Use Committee of the Fourth Hospital of Hebei Medical University (Approval number: IACUC-4th Hos Hebmu-2023214).

### 2.2 Experimental groups and methodology

#### 2.2.1 Groups

Twelve Bama miniature pigs were randomly divided into three groups of four pigs each: the 4-h compression group (Group A), the 8-h group (Group B), and the 16-h group (Group C). Each group was evaluated at five time points: baseline (T0), immediately after decompression (T1), and at 12 h (T2), 24 h (T3), and 72 h (T4) post-decompression.

#### 2.2.2 Anesthesia

The protocol described by Bunnag et al. (23) was followed, with modifications. Briefly, premedication with intramuscular atropine (0.1 mg/kg) was administered to reduce secretions. Anesthesia was then induced with IM xylazine (2.0 mg/kg) and tiletamine-zolazepam (6.0 mg/kg), and maintained with inhaled isoflurane (1–3%) in a 1:1 oxygen-nitrous oxide mixture following endotracheal intubation. Analgesia was provided by a continuous IV fentanyl infusion (5–10 μg/kg/h), supplemented by pre- and post-operative buprenorphine (0.01 mg/kg, IM). Physiological stability was ensured via continuous monitoring of ECG, heart rate, and oxygen saturation, with ventilation adjusted to maintain normal blood gas levels throughout all procedures. Throughout the 72-h post-operative observation period, animals were monitored at least every 6 h for predefined humane endpoints, including severe respiratory depression, hypothermia, or a moribund state. The deaths in Group C occurred at 48, 50, and 51 h post-decompression as acute events following a period of severe compromise, despite this monitoring schedule.

#### 2.2.3 Crush injury induction

Following surgical preparation, the experimental crush injury was induced. Animals were maintained under general anesthesia for the entire compression period. The right hindlimb was positioned on a custom-designed mechanical press equipped with a hydraulic pressure system. Compression was applied via a rectangular, flat-surfaced piston (6 cm × 4 cm, surface area 24 cm^2^) positioned perpendicularly over the mid-belly of the biceps femoris muscle. The compression surface was smooth and non-serrated to minimize skin laceration. The applied force was continuously monitored using an inline digital force gage, and the target compression force was calculated as 10× the animal’s body weight (e.g., approximately 250 kg force for a 25 kg pig). This calibrated pressure was maintained continuously for the designated duration (4, 8, or 16 h for Groups A, B, and C, respectively) via the manually controlled hydraulic system. The model was designed to produce a standardized, reproducible ischemic crush injury, consistent with the principles of large-animal trauma models (24).

#### 2.2.4 Hemodynamic monitoring and imaging

**Figure 1:**
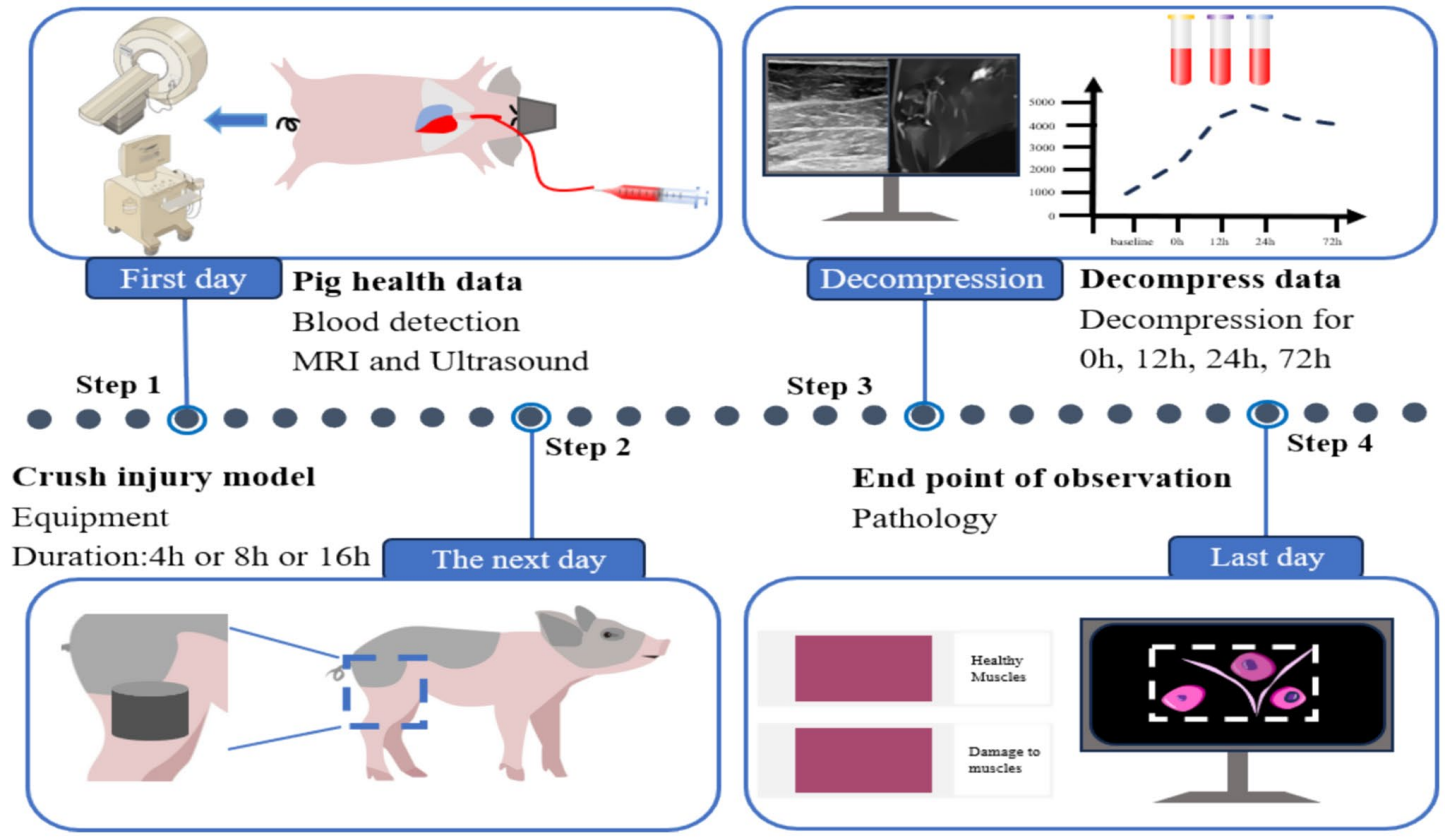
Study flowchart of porcine crush injury model showing: (1) Baseline data collection 24 h pre-experiment, (2) hindlimb compression (4/8/16 h) in three groups, (3) post-decompression monitoring at 0/12/24/72 h with biochemical/imaging data collection, and (4) terminal sampling at 72 h.

To enable hemodynamic monitoring and blood sampling, a carotid artery cannulation was performed. Throughout the experiment, biochemical indicators and imaging modalities (MRI and ultrasound) were monitored at all designated time points (Figure 1).

#### 2.2.5 Euthanasia and tissue harvest

Euthanasia and tissue collection were performed at the final time point (T4) to enable definitive pathological assessment of muscle damage. The procedure was conducted in accordance with the method reported by Jin et al. (25). Animals were maintained under a deep surgical plane of anesthesia, after which euthanasia was induced by intravenous (IV) administration of a pentobarbital sodium overdose (100 mg/kg) through a secure peripheral catheter. Bilateral thoracotomy was subsequently performed as a secondary method to ensure death. Muscle tissue from the compressed hind limb was immediately harvested. For histopathological analysis, samples were fixed in 10% neutral buffered formalin and processed for hematoxylin and eosin (H&E) staining.

### 2.3 Blood collection and analysis

Arterial blood samples were collected via carotid artery cannulation from all 12 Bama pigs at designated time points. Following centrifugation, serum was separated for analysis. We measured CK, LDH, and potassium (K^+^) levels using a fully automated biochemical analyzer (Beckman Power Express, United States).

### 2.4 Ultrasound examination

The Bama pigs were maintained under stable anesthesia throughout the procedure. Ultrasonography was performed using a Philips EPIQ 7 color Doppler system equipped with an El18-4 linear array probe (4–18 MHz frequency), acquiring high-resolution images at 3.5 cm tissue depth to assess muscle structural integrity, echo intensity variations, and textural abnormalities.

### 2.5 MRI examination

During MRI procedures, Bama pigs were maintained under continuous anesthesia while immobilized in a supine position on a custom-made, MRI-compatible plastic plate. This plate was used to ensure consistent positioning, minimize motion artifacts during scanning, and provide secure hindlimb support throughout the imaging session. Examinations were performed on a Siemens Skyra 3.0 T MRI system equipped with a 16-channel body coil, with animals positioned supine in a head-first orientation. The scanning area encompassed the region from the bilateral iliac crest to the distal hindlimbs. An oblique sagittal T2-weighted Dixon sequence was employed with the following parameters: TR 3520 ms, TE 117 ms, flip angle 123°, field of view (FOV) 380 × 333 mm, slice thickness 3.5 mm with 0.7 mm spacing, acquiring 20 total slices over approximately 2 min. These parameters, including the specific TE and flip angle, were optimized for the Dixon fat-water separation algorithm at 3.0 T to ensure robust image quality and soft tissue contrast.

#### 2.5.1 Image analysis and region of interest

For the semi-quantitative analysis, the In-Phase reconstruction of the T2-weighted Dixon sequence was used. The mean signal intensity was measured from manually drawn regions of interest (ROIs). The In-Phase images, which provide a composite water and fat signal analogous to conventional T2-weighted images, were selected for their consistent anatomical definition, enabling reliable identification of muscle boundaries and edematous regions across all time points and animals. While Water-only images offer high fluid sensitivity, they can be susceptible to artifacts at tissue interfaces; the In-Phase images provided a more stable signal baseline for longitudinal comparison.

#### 2.5.2 ROI placement protocol

For each animal at each time point, the single axial slice displaying the most extensive area of abnormal high signal intensity within the compressed biceps femoris muscle was identified by visual inspection. On this slice, a single, contiguous ROI was manually drawn to encompass the entire cross-sectional area of the muscle that demonstrated visible signal abnormality. The ROI border was carefully traced just inside the muscle fascia, extending from the femur to the subcutaneous fat layer, and deliberately excluding bone, major vessels, and intermuscular adipose tissue. The mean signal intensity (in arbitrary units) of all pixels within this entire ROI was recorded. This method provided a consistent metric reflecting the overall signal change within the affected muscle compartment. All ROI placements and signal intensity measurements were performed by a single experienced radiologist who was blinded to the experimental group and time point to minimize bias.

### 2.6 Pathological tissue analysis

Following the completion of experimental observations (post-T4 timepoint), Bama pigs were humanely euthanized under ethical guidelines. The biceps femoris muscle specimens were collected, immediately fixed in 4% paraformaldehyde, and prepared for histological processing. The tissue samples underwent paraffin embedding, sectioning, and hematoxylin–eosin (HE) staining to evaluate the severity of muscle pathology. Baseline control muscle tissue was obtained via biopsy from the right hindlimb of each animal immediately prior to compression (T0). These samples provided pre-injury normal tissue for histopathological comparison.

### 2.7 Statistical analysis

All analyses were performed using SPSS 26.0 (IBM Corp.). Normally distributed data are presented as mean ± standard deviation (x̄ ± s). Within-group comparisons utilized one-way repeated measures ANOVA, while between-group comparisons employed one-way ANOVA at T0 through T3 timepoints. Independent samples *t*-tests were conducted for T4 comparisons. Correlation analyses used Pearson coefficients, with statistical significance defined as *p* < 0.05.

#### 2.7.1 Handling of mortality and missing data

Three animals in Group C died before the final time point (T4). Therefore, all longitudinal statistical analyses were performed using only available data. For within-group comparisons over time (T0-T4), standard repeated-measures ANOVA was applied to Groups A and B, which had complete data. For Group C, due to informative missingness in mortality, a restricted analysis was performed comparing time points T0 to T3 using available data. For all between-group comparisons (A vs. B vs. C) at specific time points (T0-T3), one-way ANOVA was applied using the data from all surviving animals at that specific time point. Data from animals that died were included in the analysis for all time points prior to their death.

## 3 Results

### 3.1 General observations

All 12 Bama pigs were in good condition before extrusion, exhibiting normal movement, steady respiration, and no abnormal signs. No mortality occurred during the extrusion modeling procedure. Following decompression, groups A and B showed no fatalities, whereas 3 animals in group C died at 48 h, 50 h, and 51 h post-decompression. Post-decompression observations included depressed mental status and reduced mobility, significant localized swelling with tenderness at extrusion sites, and firm texture upon palpation. Subcutaneous bruising was also observed in some cases. The muscle swelling progressively worsened with longer extrusion times, and isolated muscle specimens exhibited dark red discoloration.

### 3.2 Time-course serum biomarker comparisons

**Figure 2:**
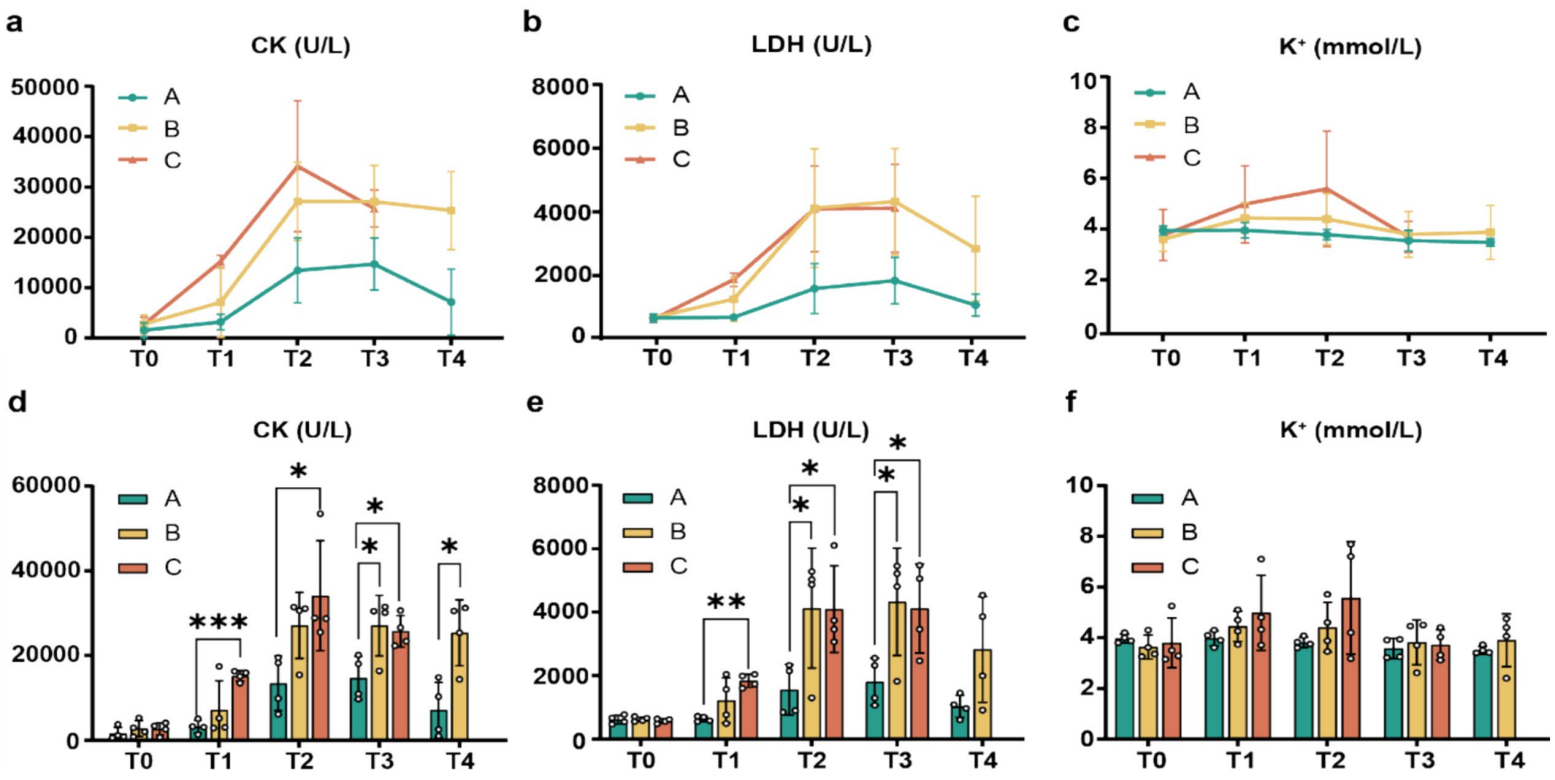
Changes in biochemical indicators across groups A, B, and C. **(a–c)** Temporal trends of CK, LDH, and K^+^ levels within each group; **(d–f)** comparative trends and statistical significance of CK, LDH, and K^+^ levels among groups (*n* = 4; *p < 0.05, **p < 0.01, ***p < 0.001).

The following analyses of biochemical markers over time include all available data, as detailed in the statistical methods. Our findings revealed significant variations in serum CK, LDH, and K + levels both within and between groups over time (Supplementary Table S1). The analysis demonstrated clear temporal patterns, with most markers showing an initial increase followed by a gradual decline. Within-group comparisons highlighted distinct trends in CK levels across all three groups. Group A exhibited significantly elevated CK at T2 and T3 compared to baseline (T0), peaking at T3. Similarly, Group B showed sustained increases from T1 through T4, reaching its highest level at T2, while Group C displayed significant rises from T1 to T3, peaking at T2 (Figure 2a).

A parallel trend was observed in LDH levels, which rose before gradually decreasing. Group A’s LDH peaked at T3, whereas Groups B and C remained significantly above baseline from T1-T4 and T1-T3, respectively, both achieving peak concentrations at T3 (Figure 2b). Interestingly, potassium (K+) dynamics varied slightly, Groups A and C showed the expected rise and fall, but Group B had a minor late increase at T4. Groups A and B reached peak K + levels at T1, while Group C’s increase was delayed, becoming significantly elevated from T2 onward and peaking at T4 (Figure 2c). Between-group comparisons yielded further insights. While no baseline (T0) differences existed, later time points revealed key divergences. For CK, Group B surpassed Group A at T3 and T4, whereas Group C consistently outperformed Group A from T1-T3 but never significantly differed from Group B. A similar pattern emerged for LDH: Group B exceeded Group A at T2-T3, and Group C remained higher than Group A from T1-T3 without differing from Group B. Notably, K+ levels showed no intergroup differences at any stage (Figures 2d–f).

### 3.3 2D ultrasound findings

**Figure 3:**
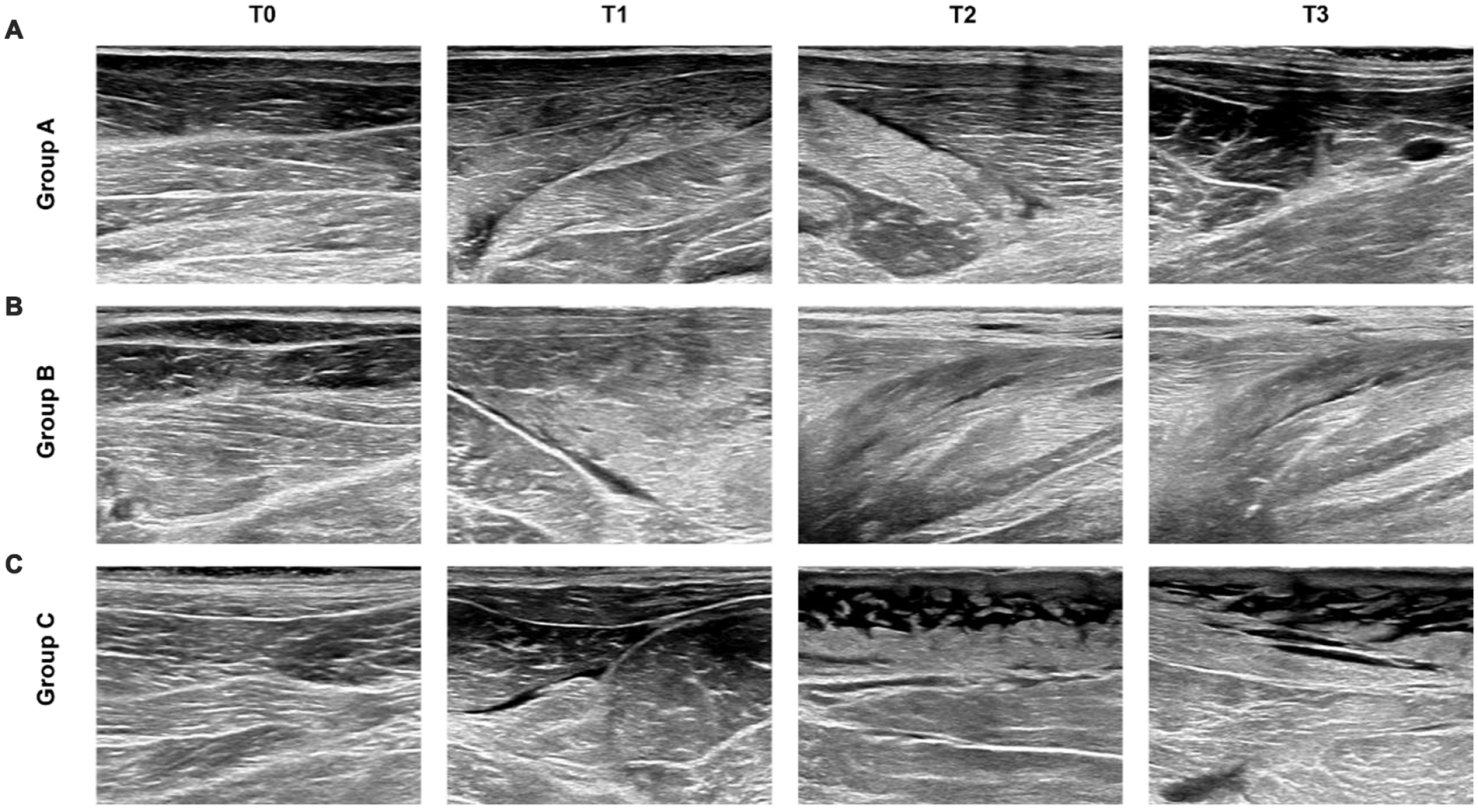
2D ultrasound findings in groups A, B, and C post-decompression. **(a)** Group A: Mild echogenicity enhancement with preserved texture, indicating gradual recovery. **(b)** Group B: Muscle thickening with blurred texture, suggesting partial recovery. **(c)** Group C: Disordered echogenicity (“sieve-like” hypoechoic, “cloudy” appearance), showing progressive worsening.

Ultrasound findings revealed a clear temporal progression of muscle changes. At T0, all groups showed well-aligned, hypoechoic muscle bundles. At T1, Group A developed mild echogenic enhancement and structural disorganization. These changes worsened at T2-T3, showing significant enhancement with irregular hypoechoic areas, before demonstrating partial recovery by T4. Group B exhibited persistent, homogeneous echogenic enhancement accompanied by complete loss of the normal fibrillar muscle architecture, resulting in a featureless appearance at all post-decompression time points, peaking at T2-T3 with slight improvement by T4. Most strikingly, Group C showed severe echogenic enhancement and textural disruption at T1, progressing to multiple irregular hypoechoic zones and fat layer thickening at T2-T3, with no meaningful recovery at T4 (Figure 3).

Figure 3 presents representative ultrasound images from T1 to T3 that capture the key diagnostic phases of injury evolution across all groups. Ultrasound images at the final T4 time point (72 h) are not displayed for the following reasons: In Group C, all animals succumbed to severe crush syndrome between 48 and 51 h post-decompression, prior to the T4 imaging time point. For Groups A and B, the T4 findings demonstrated stabilization or early resolution of changes that were consistent with the trends established at T3. Presenting the complete T1-T3 series provides a clear and comparable visual timeline of the acute and subacute injury progression across all experimental conditions.

### 3.4 Changes in MRI T2-weighted Dixon

**Figure 4:**
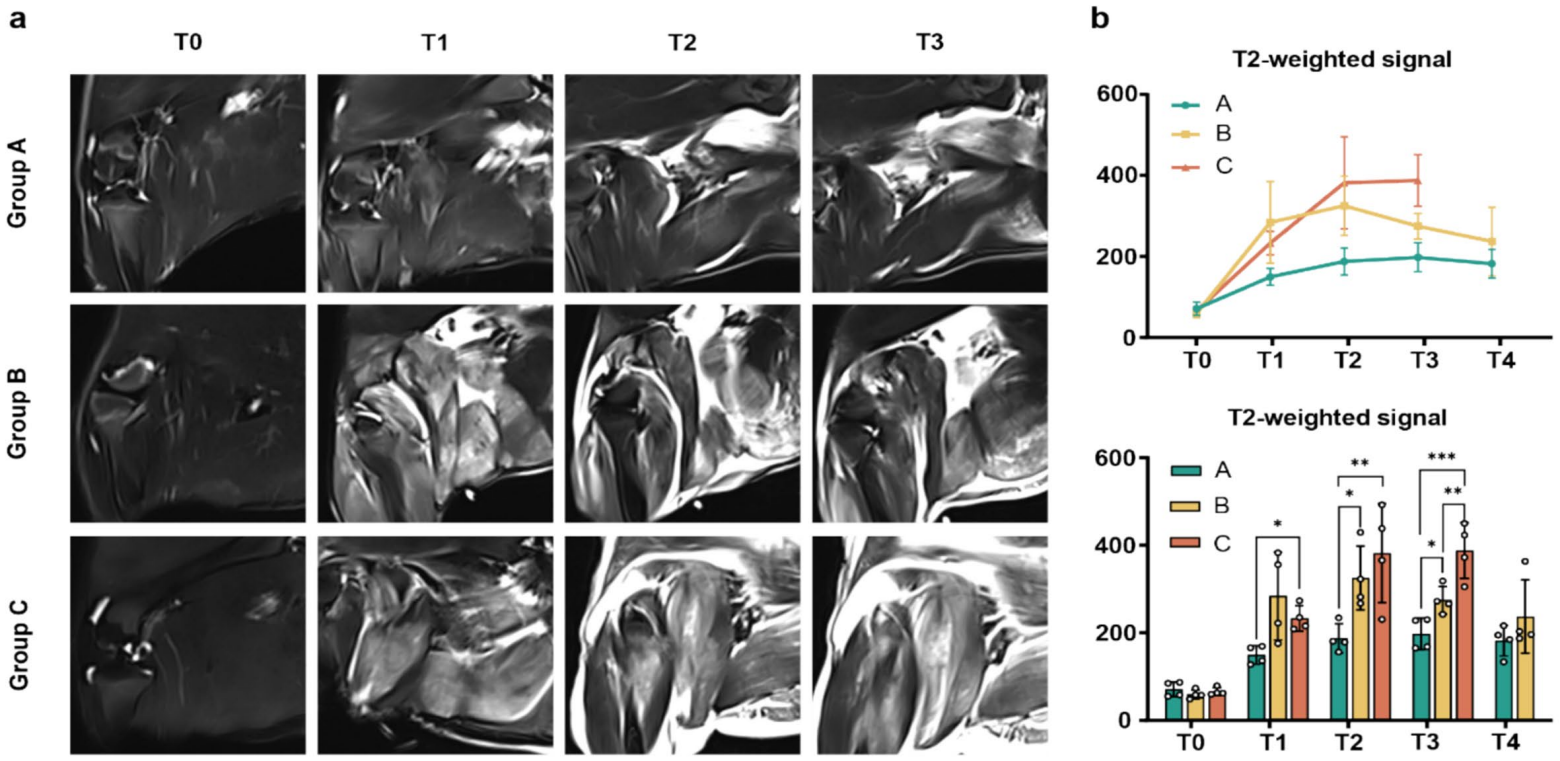
Comparison of MRI images and signal values among groups A, B, and C. **(a)** Representative MRI muscle images from experimental animals in all three groups at different time points; **(b)** Delineation of regions of interest in MRI images with analysis of signal value change trends and statistical significance within and between groups (*n* = 4; *p < 0.05, **p < 0.01, ***p < 0.001).

On T2-weighted Dixon images, all three animal groups exhibited low signal intensity at T0. Muscle fibers displayed a feathery structure with uniformly distributed delicate connective tissue, confirming intact muscle architecture and the absence of abnormal high signal. Following decompression, T2-weighted images at each subsequent time point revealed inhomogeneous high signal intensity, peaking at T2 or T3. By T4, Groups A and B showed a slight reduction in signal brightness, whereas Group C maintained elevated intensity without significant decline. The edema area and T2-weighted signal intensity progressively expanded and intensified with prolonged extrusion time across all groups (Figure 4a).

### 3.5 MRI T2-weighted signal characteristics

T2-weighted signal value analyses over time include all available data. For Group C, analysis was restricted to T0-T3 due to mortality. Semi-quantitative analysis of T2-weighted Dixon In-Phase images, measuring the mean signal intensity within a manually drawn region of interest encompassing the abnormal muscle area, revealed the following trends (Supplementary Table S2): In Group A, signal values increased significantly from T1 to T4 compared to T0, peaking at T3. Group B also showed a significant rise from T1 to T4, with peak intensity occurring earlier at T2. In Group C, values increased significantly from T1 to T3, reaching their highest point at T3. Intergroup comparisons demonstrated that Group B exhibited significantly higher signal values than Group A at T2 and T3, while Group C surpassed Group A from T1 to T3. Notably, Group C also showed significantly elevated signal intensity compared to Group B at T3 (Figure 4b).

### 3.6 Correlation analysis

**Figure 5:**
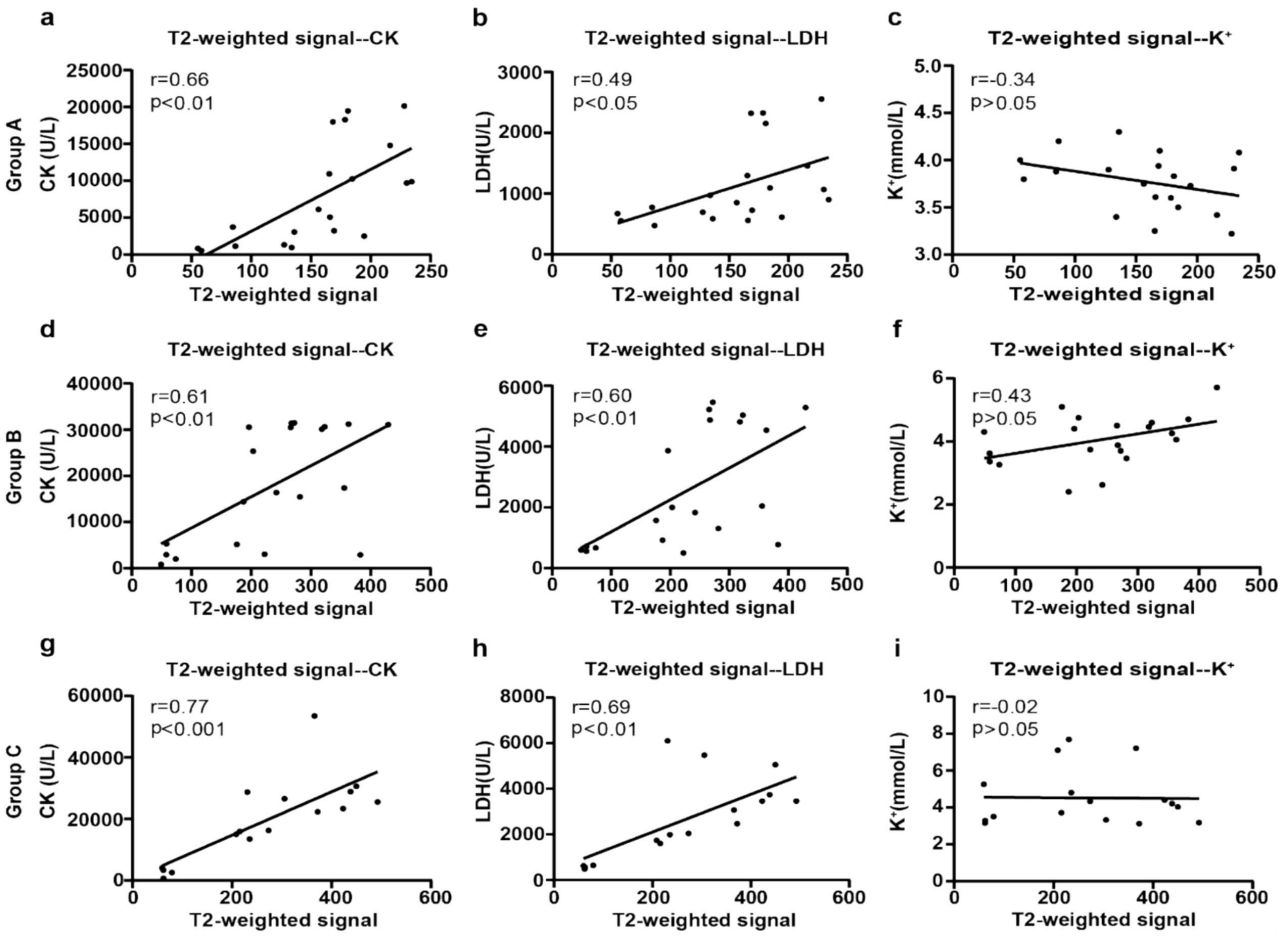
Correlations between T2WI signal values in groups A, B, and C and various biochemical indicators. **(a–c)** Correlation analyses of T2WI signal values in group A with biochemical indicators; **(d–f)** group B analyses; **(g–i)** group C analyses.

Analysis of groups A, B, and C revealed significant positive correlations between both CK and T2-weighted signal values, as well as between LDH and T2-weighted signal values (Figure 5; Supplementary Table S3). In Group A, there was a moderate correlation for CK (*r* = 0.66, *p* < 0.01) and a weak correlation for LDH (*r* = 0.49, *p* < 0.05). Group B showed moderate correlations for both CK (*r* = 0.61, *p* < 0.01) and LDH (*r* = 0.60, *p* < 0.01). Consistent with the graphical data, Group C demonstrated the strongest correlations, with a strong positive correlation for CK (*r* = 0.77, *p* < 0.001) and a moderate correlation for LDH (*r* = 0.69, *p* < 0.01). However, no significant correlation was observed between potassium ion levels (K^+^) and T2-weighted signal values in any group (all *p* > 0.05).

### 3.7 Experimental muscle pathology

**Figure 6:**
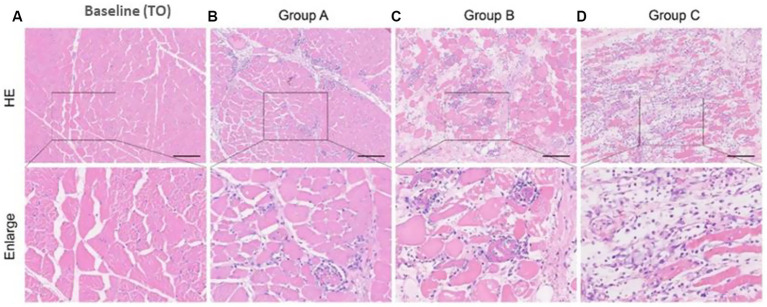
Representative hematoxylin and eosin (H&E) stained muscle sections. **(a)** Baseline (T0): Normal muscle architecture before compression. **(b)** Group A (72 h post-decompression): Mild pathological changes. **(c)** Group B (72 h post-decompression): Moderate damage with inflammatory infiltration. **(d)** Group C (at death, ~48–51 h post-decompression): Severe necrosis and architectural disruption.

The histological examination revealed progressive muscle damage across the experimental groups. Muscle sections from the pre-compression baseline (T0) showed structurally intact, regularly arranged muscle fibers. Group A exhibited mild pathological changes characterized by some fiber detachment and swelling, though the overall structure remained preserved mainly. More severe damage was observed in Group B, where muscle fibers appeared irregularly arranged with marked swelling and increased inflammatory cell infiltration. The most extensive damage occurred in Group C, featuring myofiber breaks, necrotic cells with vacuolization, cytoplasmic homogenization, and prominent inflammatory cell infiltration. Specimens for Group C were collected at the time of animal death (48–51 h post-decompression) (Figure 6). The baseline (T0) histology is included in Figure 6 for reference.

## 4 Discussion

To address the translational limitations of small animal models in crush injury (CI) research (25), we established a porcine model that simulates prolonged muscle compression. This large-animal model enabled, for the first time, longitudinal imaging of dynamic pathological changes, providing a more clinically relevant platform. Our approach facilitates more reliable correlation between imaging parameters and biochemical markers, thereby enhancing the potential for clinical translation.

Biochemical markers (CK, LDH, and K^+^) are established indicators of muscle injury. While myoglobin is a sensitive early marker, we focused on CK and LDH due to CK’s role as the cornerstone biomarker for rhabdomyolysis, offering high specificity for muscle injury and a longer half-life that is more reliable for monitoring injury progression beyond the initial hours. In this study, CK levels in all groups exhibited a rise-and-fall pattern proportional to compression duration, peaking earlier (12–24 h) in severe CI, consistent with prior reports (26, 27). Groups A and B showed CK normalization by T3, suggesting reversible damage after short compression, while Group C’s sustained elevation indicated profound, potentially irreversible injury. LDH followed similar trends but with lower sensitivity. K^+^ spiked earliest across groups, reflecting rapid cellular rupture (28), highlighting its diagnostic value for early CI detection. These findings align biochemical dynamics with injury severity and duration, offering insights for clinical assessment.

This study pioneered the application of T2-weighted Dixon MRI and 2D ultrasound for dynamic evaluation of CI in a large animal model. Both imaging modalities effectively captured temporal changes at injury sites. T2-weighted Dixon images revealed inhomogeneous high signal intensities post-decompression, indicative of muscle edema, extracellular fluid accumulation, and microhemorrhage. The acute phase (T1-T2) showed significant signal elevation, reflecting acute edema and inflammatory responses. During the intermediate phase (T2-T3), expanding high-signal regions suggested persistent inflammation and fluid retention. By T4, slightly diminished signals in Groups A and B indicated partial edema resolution, while Group C maintained elevated signals due to prolonged compression, suggesting ongoing pathology. These findings align with prior rabbit CI studies (29), demonstrating T2-weighted Dixon’s utility for early CI detection, longitudinal monitoring, and repair assessment, as it accurately mirrors the pathological progression of muscle edema and necrosis.

Further analysis revealed significant positive correlations between both CK and T2-weighted signal values and between LDH and T2-weighted signal values across all groups, with the strongest correlations observed in Group C. In contrast, no significant correlation was found between K^+^ levels and T2-weighted signals. These findings demonstrate the complementary relationship between biochemical and imaging markers. While CK serves as a classical indicator of muscle injury severity through myocyte membrane damage, T2-weighted signals primarily reflect edema and extracellular fluid accumulation. The robust correlation in Group C, which experienced prolonged compression, suggests that imaging features are susceptible to biochemical changes in severe injuries. Similarly, the LDH-T2 signal correlation supports imaging’s utility in assessing cellular necrosis and inflammatory responses, with Group C’s results highlighting its potential as a synchronous indicator of injury extent. The lack of K^+^ correlation likely reflects its early release during initial cellular rupture, while semi-quantitative T2-weighted signals track later-phase edema and inflammation. This distinction indicates that while K^+^ has value for early diagnosis, it offers limited monitoring capability compared to the combined use of T2-weighted imaging with CK and LDH. Together, these modalities provide a more complete assessment of injury progression (30).

The 2D ultrasound effectively captured dynamic structural and echogenic changes in muscle tissue throughout the observation period. Following decompression, T1 images revealed early edema and extracellular fluid accumulation, while T2-T3 demonstrated marked echo enhancement, indicating necrotic expansion. By T4, Groups A and B exhibited slight signal reduction, suggesting tissue recovery, whereas Group C maintained disorganized echotexture, reflecting severe, persistent damage. These ultrasound findings correlated strongly with biochemical markers, offering a reliable visual assessment of injury severity and repair progression in crush injury. The technique’s high resolution, real-time capability, and operational efficiency underscore its clinical utility for monitoring muscle injury recovery (31). In this study, we employed descriptive, qualitative ultrasound to provide a real-time, high-resolution anatomical correlate to the semi-quantitative MRI and biochemical data. This approach was chosen to capture the complex spectrum of textural changes (e.g., loss of architecture, heterogeneous echogenicity) that are readily appreciable by a trained clinician but are not fully captured by a single quantitative metric, thereby serving as a complementary diagnostic tool for initial assessment and monitoring.

The combined use of T2-weighted Dixon MRI and 2D ultrasound offers a pragmatic solution for crush injury assessment, effectively balancing diagnostic depth with clinical feasibility. While the T2-weighted Dixon sequence provides rapid, sensitive detection of muscle pathology, it is crucial to acknowledge the inherent limitations of MRI in acute trauma settings. These include prolonged scan times, limited scanner availability in remote or disaster-stricken areas, and the practical challenges of managing unstable, polytrauma patients within the MRI environment (7). It is precisely these constraints that underscore the complementary value of ultrasound. Ultrasound delivers high-resolution, real-time structural evaluation at the point-of-care, overcoming the logistical barriers of MRI. This dual-imaging paradigm suggests a practical diagnostic strategy: ultrasound can serve as a tool for rapid triage and monitoring, while Dixon MRI, when logistically feasible, can provide a more comprehensive assessment to inform management. This multimodal approach could help overcome the limited specificity of biochemical markers and offer a more adaptable framework for CI assessment across various clinical settings.

### 4.1 Limitations and future directions

This study has several limitations: (1) the small sample size may limit result generalizability; (2) while using a large animal model, interspecies differences between porcine and human pathophysiology remain, requiring future clinical validation; (3) the imaging analysis has inherent qualitative components. The T2-weighted Dixon analysis provided semi-quantitative signal intensity, while the ultrasound assessment was descriptive. The future incorporation of quantitative ultrasound techniques (e.g., elastography, grayscale histogram analysis) or a validated grading system would enhance objectivity and reproducibility; (4) while a standardized anesthetic protocol was used, the potential influence of fentanyl on hemodynamics and the absence of continuous blood pressure monitoring should be considered, as these factors may have affected muscle perfusion and subsequent biochemical or imaging findings; (5) the semi-quantitative MRI analysis relied on single-observer ROI placement. Although performed by a blinded, experienced radiologist, formal reproducibility analysis (e.g., intra- and inter-observer correlation) was not conducted; and (6) The death of three animals in the severe injury group (C) raises an important ethical consideration. In retrospect, the definition of a humane endpoint for this severe model should have been more conservative to require euthanasia prior to the point of spontaneous death. Future studies will implement stricter, earlier clinical endpoints (e.g., predefined severe score on a validated animal health scale) and more frequent monitoring in severe injury groups to ensure immediate euthanasia is performed if such endpoints are reached. Future studies should incorporate quantitative techniques such as T2 mapping and ultrasound elastography to enhance the precision of injury assessment.

## 5 Conclusion

This study demonstrates the innovative application of T2-weighted Dixon MRI and 2D ultrasound for dynamic assessment of crush injury (CI) in a large animal model, providing the first sequential imaging evidence correlating with biochemical marker progression. The T2-weighted Dixon sequence’s sensitivity to edema and necrosis complements ultrasound’s high-resolution real-time imaging, establishing a comprehensive multimodal platform that overcomes the limitations of biochemical markers alone. This integrated approach offers precise CI evaluation to guide clinical decision-making and enhance treatment outcomes.

## Data Availability

The raw data supporting the conclusions of this article will be made available by the authors, without undue reservation.
