# Supplementary material for: Dynamic muscle damage monitoring in pig crush injury: T2-weighted Dixon and 2D ultrasound applications
Source: Front Vet Sci. 2026 Feb 25;13:1692050. doi: 10.3389/fvets.2026.1692050 (PMC12975460; doi:10.3389/fvets.2026.1692050)
Supplement: Supplementary file 1 [file Table_1.docx]

**Dynamic Muscle Damage Monitoring in Pig Crush Injury: T2-Weighted Dixon and 2D Ultrasound Applications**

**Supplementary data**

**Table S1.** Changes within and between groups for each biochemical indicator.

| Group | T0 | T1 | T2 | T3 | T4 |
| --- | --- | --- | --- | --- | --- |
| CK  (U/L) |  |  |  |  |  |
| A | 1566.20  ±1463.49 | 3191.75  ±1535.62 | 13442.20  ±6473.09^☆^ | 14695.50  ±5148.20^☆^ | 7134.33  ±6532.64 |
| B | 2750.10  ±1887.81 | 7115.58  ±6934.10^☆^ | 27142.28  ±7803.24^☆^ | 27120.30  ±7175.59^☆★^ | 25371.03  ±7772.11^☆★^ |
| C | 2672.95  ±1452.80 | 15190.55  ±1242.26^☆▲^ | 34144.60  ±12988.44^☆▲^ | 25731.65  ±3726.32^☆▲^ | - |
| LDH  (U/L) |  |  |  |  |  |
| A | 617.25  ±132.79 | 641.00  ±81.43 | 1557.75  ±791.24 | 1810.00  ±737.88 | 1033.25  ±347.74 |
| B | 631.50  ±71.43 | 1220.50  ±712.79^☆^ | 4121.25  ±1886.96^☆★^ | 4328.25  ±1689.53^☆★^ | 2827.50  ±1666.69^☆^ |
| C | 576.75  ±70.56 | 1843.25  ±210.00^☆▲^ | 4095.25  ±1364.96^☆▲^ | 4115.50  ±1398.57^☆▲^ | - |
| K^+^  (mmol/L) |  |  |  |  |  |
| A | 3.97  ±0.17 | 3.98  ±0.29 | 3.82  ±0.20 | 3.58  ±0.40 | 3.51  ±0.15 |
| B | 3.64  ±0.47 | 4.45  ±0.58 | 4.41  ±0.98 | 3.82  ±0.88 | 3.90  ±1.04 |
| C | 3.80  ±0.98 | 4.99  ±1.48 | 5.57  ±2.22^☆^ | 3.72  ±0.60 | - |

**Note:** ☆indicates significant differences compared to the healthy group within each time period; ★indicates significant differences between Group B and Group A within the same time period; ▲indicates significant differences between Group C and Group A within the same time period.

**Data Note:** Data are presented as mean ± SD for all surviving animals at each time point. For Group C, three animals died before T4; therefore, no T4 data are available. Data from these animals are included for time points T0-T3.

**Table S2.** Changes within and between groups of T2WI signal values.

| Group | T0 | T1 | T2 | T3 | T4 |
| --- | --- | --- | --- | --- | --- |
| T2WI (Signal value) |  |  |  |  |  |
| A | 71.28  ±16.72 | 149.73  ±21.04^☆^ | 187.50  ±33.05^☆^ | 197.98  ±35.91^☆^ | 182.25  ±34.91^☆^ |
| B | 59.65  ±10.30 | 284.13  ±100.54^☆^ | 325.28  ±73.17^☆★^ | 274.63  ±31.74^☆★^ | 237.40  ±84.00^☆^ |
| C | 65.98  ±9.04 | 233.30  ±29.04^☆★^ | 381.80  ±113.26^☆★^ | 387.68  ±63.62^☆★▲^ | - |

Note: ^☆^indicates significant differences compared to the healthy group within each time period; ^★^indicates significant differences between Group B and Group A within the same time period; ^▲^indicates significant differences between Group C and Group A within the same time period.

**Data Note:** Data are presented as mean ± SD for all surviving animals at each time point. For Group C, three animals died before T4; therefore, no T4 data are available. Data from these animals are included for time points T0-T3.

**Table S3**. Correlation between T2WI signal value and various biochemical indicators.

| PCC of Groups | CK/T2WI (Signal value) | LDH/T2WI (Signal value) | K^＋^/T2WI (Signal value) |
| --- | --- | --- | --- |
| A |  |  |  |
| *r* | 0.66 | 0.49 | -0.34 |
| *p* | ＜0.01 | ＜0.05 | ＞0.05 |
| B |  |  |  |
| *r* | 0.61 | 0.60 | 0.43 |
| *p* | ＜0.01 | ＜0.01 | ＞0.05 |
| C |  |  |  |
| *r* | 0.77 | 0.69 | -0.02 |
| *p* | ＜0.001 | ＜0.01 | ＞0.05 |

Note: PCC: Pearson correlation coefficient. When 0 ≤ |r| < 0.3, it indicates no correlation; when 0.3 ≤ |r| < 0.5, it represents weak correlation; when 0.5 ≤ |r| < 0.8, it denotes moderate correlation; and when 0.8 ≤ |r| < 1, it signifies strong correlation.

**Data Note:** Data are presented as mean ± SD for all surviving animals at each time point. For Group C, three animals died before T4; therefore, no T4 data are available. Data from these animals are included for time points T0-T3.
